# Supplementary material for: Association between Plant-Based Dietary Patterns and Risk of Cardiovascular Disease: A Systematic Review and Meta-Analysis of Prospective Cohort Studies
Source: Nutrients. 2021 Nov 5;13(11):3952. doi: 10.3390/nu13113952 (PMC8624676; doi:10.3390/nu13113952)
Supplement: Supplementary file 1 [file nutrients-13-03952-s001.zip › nutrients-1426914-supplementary.pdf]

Supplementary Material

Association between plant-based dietary patterns and risk of cardiovascular disease: a systematic review and meta-analysis of prospective cohort studies

Zuo Hua Gan, Huey Chiat Cheong, Yu-Kang Tu, Po-Hsiu Kuo

Content

Supplemental Table S1. Full search strategy 2

Supplemental Table S2. Inclusion and exclusion criteria for study selection 4

Supplemental Figure S1. Forest plot of the adjusted relative risk (RR) of plant-based diet and (A) ischemic and (B) hemorrhagic stroke risks. 5

Supplemental Table S3. Subgroup analyses of the association between plant-based diet and cardiovascular disease, coronary heart disease and stroke risk 6

Supplemental Table S4. Slope (β), 95% CI, and P Values for Meta-Regression Models 9

Supplemental Figure S2. Funnel plot of the relative risk ratio (RR) of plant-based diet and (A) cardiovascular disease, (B) coronary heart disease and (C) stroke risks 10

Supplemental Figure S3. Forest plot of the adjusted relative risk (RR) of plant-based diet and (A) cardiovascular disease and (B) stroke risks with included studies and imputed studies with the trim-and-fill approach. 12

Supplemental Table S5. Quality assessment of included studies. 13

Supplemental Figure S4. Forest plot of the adjusted relative risk (RR) of (A) cardiovascular disease, (B) coronary heart disease and (C) stroke risks for highest versus lowest adherence to plant-based dietary patterns with sensitivity analyses 15

Correspondence: Po-Hsiu Kuo, phkuo@ntu.edu.tw; Institute of Epidemiology and Preventive Medicine, National Taiwan University, Room 501, No. 17, Xu-Zhou Road, Taipei 100, Taiwan.

Supplemental Table S1. Full search strategy

**PubMed**
(“plant-based” OR “plant-based diet” OR ("diet, vegetarian"[MeSH Terms]) OR ("diet"[All Fields] AND "vegetarian"[All Fields]) OR "vegetarian diet"[All Fields] OR ("diet"[All Fields] AND "vegetarian"[All Fields]) OR "diet, vegetarian"[All Fields]) OR ("diet, vegetarian"[MeSH Terms] OR ("diet"[All Fields] AND "vegetarian"[All Fields]) OR "vegetarian diet"[All Fields] OR ("vegetarian"[All Fields] AND "diets"[All Fields]) OR "vegetarian diets"[All Fields]) OR ("diet, vegetarian"[MeSH Terms] OR ("diet"[All Fields] AND "vegetarian"[All Fields]) OR "vegetarian diet"[All Fields] OR ("vegetarian"[All Fields] AND "diet"[All Fields])) OR ("diet, vegetarian"[MeSH Terms] OR ("diet"[All Fields] AND "vegetarian"[All Fields]) OR "vegetarian diet"[All Fields] OR "vegetarianism"[All Fields]) OR ("diet, vegan"[MeSH Terms] OR ("diet"[All Fields] AND "vegan"[All Fields]) OR "vegan diet"[All Fields] OR ("diet"[All Fields] AND "vegan"[All Fields]) OR "diet, vegan"[All Fields]) OR "vegetarian diet"[All Fields] OR ("vegans"[MeSH Terms] OR "vegans"[All Fields]) OR ("vegetarians"[MeSH Terms] OR "vegetarians"[All Fields])) **AND**<br>(‘cardiovascular disease’[All Fields] OR ‘cardiovascular diseases’[MeSH Terms] OR ‘coronary artery disease’[All Fields] OR ‘myocardial infarction’[MeSH Terms] OR (‘myocardial’[All Fields] AND ‘infarction’[All Fields]) OR ‘myocardial infarction’[All Fields]) OR (‘stroke’[MeSH Terms] OR ‘stroke’[All Fields]) OR ‘cerebrovascular disease’[All Fields]) **AND**<br>("observational study"[tiab] OR "observational studies"[tiab] OR "cohort studies"[MeSh] OR "cohort studies"[tiab] OR "case-control studies"[MeSh] OR "case-control studies"[tiab] OR "cross-sectional studies"[MeSh] OR "cross-sectional studies"[tiab] OR "epidemiologic studies"[MeSh] OR "epidemiologic studies"[tiab] OR "follow-up studies"[MeSh] OR "follow-up studies"[tiab] OR "longitudinal studies"[MeSh] OR "longitudinal studies"[tiab] OR "prospective studies"[MeSh] OR "prospective studies"[tiab] OR "retrospective studies"[MeSh] OR "retrospective studies"[tiab] OR "cohort study"[tiab] OR "case-control study"[tiab] OR "cross-sectional study"[tiab] OR "epidemiologic study"[tiab] OR "follow-up study"[tiab] OR "longitudinal study"[tiab] OR "prospective study"[tiab] OR "retrospective study"[tiab]))
**EMBASE**
(‘plant-based’ OR ‘plant diet’ OR ‘plant-based diet’ OR ('vegetarian'/exp OR 'vegetarian') AND ('diets'/exp OR 'diets') OR 'vegetarian diets' OR (('vegetarian'/exp OR 'vegetarian') AND ('diet'/exp OR 'diet')) OR 'diet, vegetarian'/exp OR 'diet, vegetarian' OR (('diet'/exp OR 'diet') AND ('vegetarian'/exp OR 'vegetarian')) OR 'vegetarianism'/exp OR 'vegetarianism' OR 'vegan diet'/exp OR 'vegan diet' OR (('diet'/exp OR 'diet') AND ('vegan'/exp OR 'vegan')) OR 'diet, vegan'/exp OR 'diet, vegan' OR 'vegetarian diet'/exp OR 'vegetarian diet' OR 'vegans'/exp OR 'vegans' OR 'vegetarians'/exp OR 'vegetarians') **AND**<br>((‘cardiovascular disease’ OR ‘cardiovascular disease’/exp OR ‘myocardial infarction’ OR (‘myocardial’ AND ‘infarction’) OR ‘myocardial infarction’/exp OR ‘cerebrovascular disease’ OR ‘cerebrovascular disease’/exp OR ‘stroke’ OR ‘stroke’/exp) **AND**<br>(observational studies or ‘observational study’/exp or Cohort studies or ‘cohort analysis’/exp or case control studies or ‘case control study’/exp or cross sectional studies or ‘cross-sectional study’/exp or epidemiologic studies or ‘epidemiology’/exp or follow-up studies or ‘follow up’/exp or longitudinal studies or ‘longitudinal study’/exp or prospective studies or ‘prospective study’/exp or retrospective studies or ‘retrospective study’/exp))
**Web of Science**

TS = (plant-based OR plant-based diet OR vegetarian OR vegetarianism OR vegan) **AND**
TS = (cardiovascular disease OR myocardial infarction OR cerebrovascular disease) **AND**
TS = (observational OR cohort OR case-control OR cross-sectional OR epidemiologic OR follow-up OR longitudinal OR prospective OR retrospective)

Supplemental Table S2. Inclusion and exclusion criteria for study selection

	Inclusion criteria	Exclusion criteria
**Study type**	Prospective cohort, case-cohort studies, or nested case-control studies	Retrospective case-control studies, cross-sectional studies, literature reviews and meta-analyses, commentaries, editorials, letters, case reports, and meeting abstracts
**Study population**	Healthy adults, at-risk adults	Children, adults with previous major cardiovascular diseases
**Exposure**	Plant-based dietary patterns (avoidance of animal foods as assessed by questionnaire, a priori or posteriori dietary patterns)	Unclear definitions of dietary exposure or measurements
**Outcome**	Multivariate adjusted effect estimates (OR, RR, HR)<br>1. Total cardiovascular disease (CVD)<br>2. Fatal coronary heart disease (CHD)<br>3. Nonfatal CHD<br>4. Total stroke<br>5. Fatal stroke<br>6. Nonfatal stroke<br>7. Ischemic stroke<br>8. Hemorrhagic stroke	Crude effect estimates only

(A)

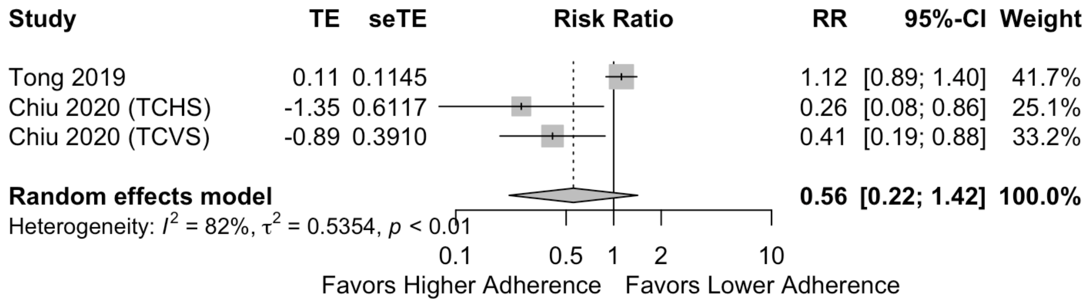

(B)

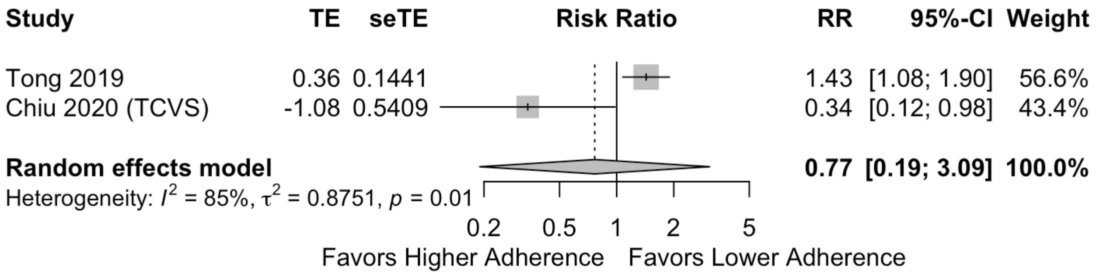

Supplemental Figure S1. Forest plot of the adjusted relative risk (RR) of plant-based diet and (A) ischemic and (B) hemorrhagic stroke risks.

Supplemental Table S3. Subgroup analyses of the association between plant-based diet and cardiovascular disease, coronary heart disease and stroke risk

Subgroups	No. of studies	RR (95% CI)	Heterogeneity between study, I^2, p value	Test for subgroup differences, p value
**Cardiovascular disease**				
Sex				0.17
Male	3	0.88 (0.83, 0.94)	12%, 0.32	
Female	4	0.80 (0.71, 0.91)	83%, <0.01	
Mean age				
>50	6	0.88 (0.84, 0.92)	36%, 0.17	< 0.01
⩽50	3	0.73 (0.63, 0.84)	41%, 0.18	
Country				0.05
USA	5	0.84 (0.79, 0.91)	56%, 0.06	
UK	2	0.85 (0.73, 0.99)	84%, 0.01	
Taiwan	2	0.52 (0.36, 0.76)	0%, 0.96	
Follow up duration				0.76
≤20 years	5	0.82 (0.70, 0.96)	72%, <0.01	
>20 years	4	0.84 (0.78, 0.90)	61%, 0.05	
Diet classification method				0.30
Questionnaire	4	0.78 (0.65, 0.93)	78%, <0.01	
A priori	4	0.84 (0.78, 0.90)	61%, 0.05	
A posteriori	1	1.02 (0.76, 1.36)	-	
Outcome				0.04
CVD	4	0.86 (0.80, 0.92)	71%, 0.01	
CHD	3	0.83 (0.75, 0.92)	37%, 0.20	
CVA	2	0.52 (0.36, 0.76)	0%, 0.96	
Study quality scores				0.03
7	3	0.66 (0.48, 0.90)	51%, 0.13	
8	4	0.84 (0.78, 0.90)	61%, 0.05	
9	2	0.91 (0.87, 0.96)	0%, 0.45	
**Coronary heart disease**				
Sex				0.55
Male	3	0.89 (0.79, 1.01)	34%, 0.22	
Female	4	0.82 (0.67, 1.01)	73%, 0.01	
Mean age				
>50	5	0.93 (0.87, 1.00)	0%, 0.66	< 0.01

Subgroups	No. of studies	RR (95% CI)	Heterogeneity between study, I^2, p value	Test for subgroup differences, p value
≤50	1	0.78 (0.70, 0.87)	-	
Country				0.61
USA	4	0.92 (0.83, 1.01)	0%, 0.56	
UK	2	0.86 (0.71, 1.06)	85%, 0.01	
Follow up duration				0.54
≤20 years	4	0.91 (0.80, 1.03)	67%, 0.03	
>20 years	2	0.85 (0.73, 0.99)	0%, 0.55	
Diet classification method				0.66
Questionnaire	2	0.86 (0.71, 1.06)	85%, 0.01	
A priori	3	0.90 (0.81, 0.94)	0%, 0.48	
A posteriori	1	1.02 (0.76, 1.36)	-	
Healthful plant-based diet				0.32
Yes	5	0.87 (0.78, 0.96)	41%, 0.15	
No	5	0.86 (0.79, 0.92)	27%, 0.22	
Study quality scores				0.02
7	1	0.78 (0.70, 0.87)	-	
8	3	0.90 (0.82, 1.00)	0%, 0.48	
9	2	0.97 (0.87, 1.08)	0%, 0.70	
**Stroke**				
Country				0.01
USA	2	0.91 (0.89, 0.93)	0%, 0.62	
UK	2	1.02 (0.72, 1.44)	83%, 0.02	
Taiwan	2	0.52 (0.35, 0.76)	0%, 0.96	
Follow up duration				0.06
≤20 years	3	0.65 (0.45, 0.96)	53%, 0.12	
>20 years	3	0.98 (0.80, 1.19)	83%, <0.01	
Diet classification method				0.67
Questionnaire	4	0.78 (0.53, 1.15)	84%, <0.01	
A priori	1	0.91 (0.89, 0.93)	-	
A posteriori	1	0.85 (0.65, 1.12)	-	
Study quality scores				0.57
7	3	0.72 (0.36, 1.41)	87%, 0.01	
8	1	0.91 (0.89, 0.93)	-	
9	2	0.84 (0.70, 1.01)	0%, 0.95	

The summary RR estimates were calculated using the random-effects models. The reference was the lowest adherence to the plant-based diet category

Supplemental Table S4. Slope (β), 95% CI, and P Values for Meta-Regression Models

	CVD risk		CHD risk		Stroke risk	
	β, 95% CI	P value	β, 95% CI	P value	β, 95% CI	P value
Mean age at baseline (years)	0.013 (0.006, 0.019)	< 0.001	0.016 (0.005, 0.027)	0.006	-0.009 (-0.041, 0.024)	0.594
Mean BMI at baseline	0.029 (-0.007, 0.065)	0.109	0.056 (0.018, 0.093)	0.004	-0.053 (-0.128, 0.023)	0.170
Female proportion (%)	-0.001 (-0.003, 0.000)	0.109	-0.002 (-0.004, 0.001)	0.175	0.013 (-0.008, 0.033)	0.237
Length of follow up (years)	-0.002 (-0.009, 0.006)	0.648	-0.005 (-0.015, 0.005)	0.319	0.011 (-0.015, 0.046)	0.409
Smokers proportion (%)	-0.002 (-0.013, 0.009)	0.744	-0.004 (-0.022, 0.016)	0.763	0.001 (-0.017, 0.020)	0.903

Abbreviation: BMI, Body Mass Index; CHD, Coronary heart disease; CVD, Cardiovascular disease

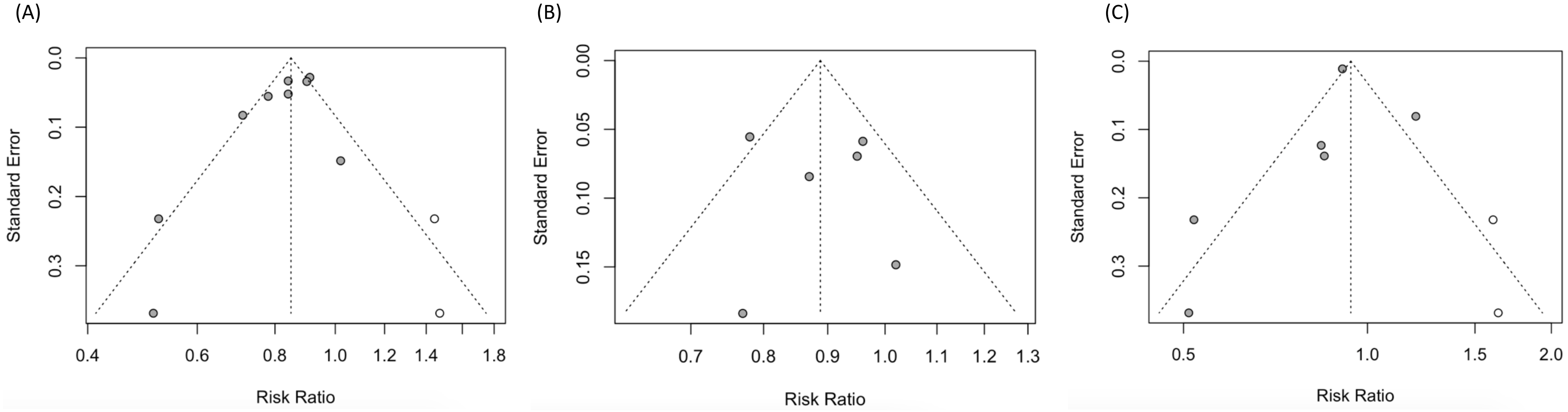

Supplemental Figure S2. Funnel plot of the relative risk ratio (RR) of plant-based diet and (A) cardiovascular disease, (B) coronary heart disease and (C) stroke risks. Gray circles = included studies; white circles = imputed studies with the trim-and-fill approach.

(A)

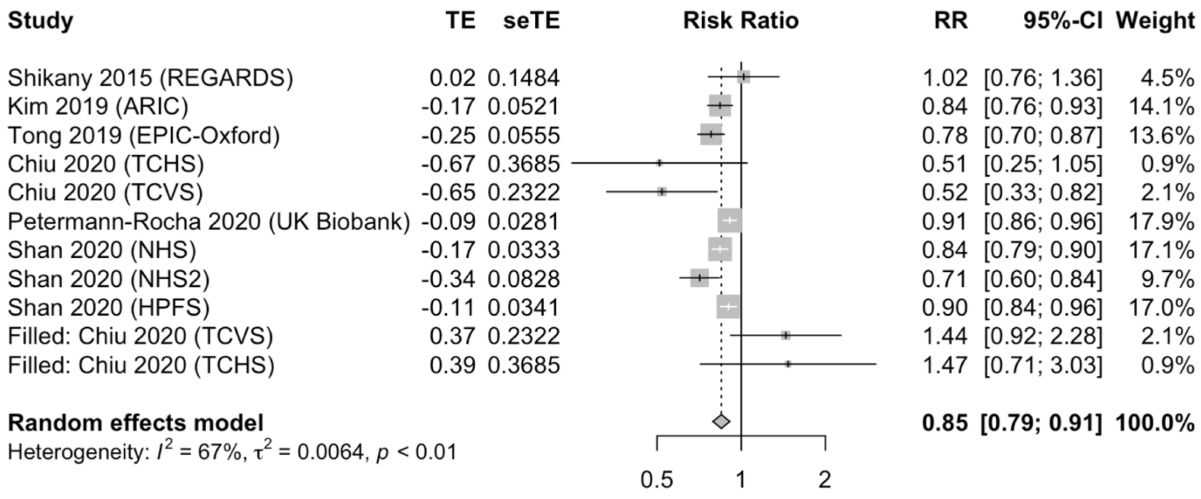
Study TE seTE Risk Ratio RR 95%-CI Weight
Shikany 2015 (REGARDS) 0.02 0.1484 1.02 [0.76; 1.36] 4.5%
Kim 2019 (ARIC) -0.17 0.0521 0.84 [0.76; 0.93] 14.1%
Tong 2019 (EPIC-Oxford) -0.25 0.0555 0.78 [0.70; 0.87] 13.6%
Chiu 2020 (TCHS) -0.67 0.3685 0.51 [0.25; 1.05] 0.9%
Chiu 2020 (TCVS) -0.65 0.2322 0.52 [0.33; 0.82] 2.1%
Petermann-Rocha 2020 (UK Biobank) -0.09 0.0281 0.91 [0.86; 0.96] 17.9%
Shan 2020 (NHS) -0.17 0.0333 0.84 [0.79; 0.90] 17.1%
Shan 2020 (NHS2) -0.34 0.0828 0.71 [0.60; 0.84] 9.7%
Shan 2020 (HPFS) -0.11 0.0341 0.90 [0.84; 0.96] 17.0%
Filled: Chiu 2020 (TCVS) 0.37 0.2322 1.44 [0.92; 2.28] 2.1%
Filled: Chiu 2020 (TCHS) 0.39 0.3685 1.47 [0.71; 3.03] 0.9%
Random effects model 0.85 [0.79; 0.91] 100.0%
Heterogeneity: I^2 = 67%, τ^2 = 0.0064, p < 0.01
0.5
1
2

(B)

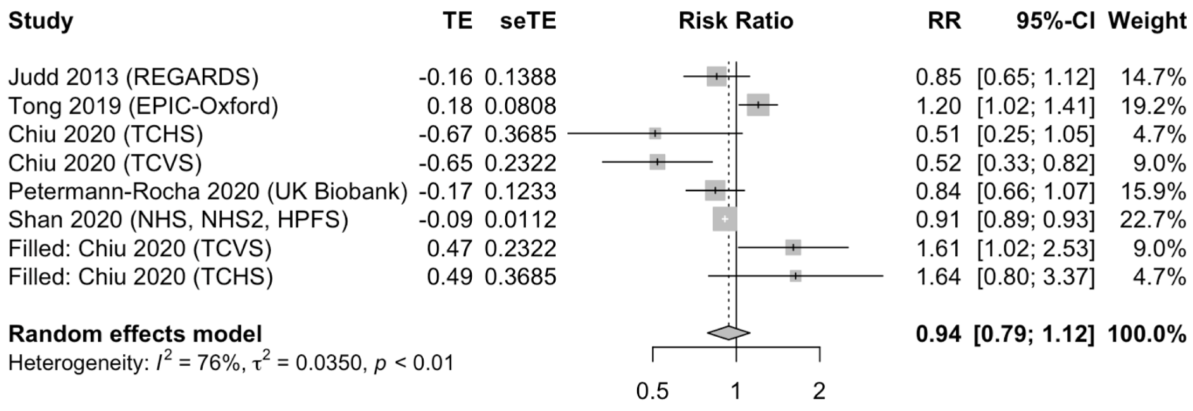
Study TE seTE Risk Ratio RR 95%-CI Weight
Judd 2013 (REGARDS) -0.16 0.1388 0.85 [0.65; 1.12] 14.7%
Tong 2019 (EPIC-Oxford) 0.18 0.0808 1.20 [1.02; 1.41] 19.2%
Chiu 2020 (TCHS) -0.67 0.3685 0.51 [0.25; 1.05] 4.7%
Chiu 2020 (TCVS) -0.65 0.2322 0.52 [0.33; 0.82] 9.0%
Petermann-Rocha 2020 (UK Biobank) -0.17 0.1233 0.84 [0.66; 1.07] 15.9%
Shan 2020 (NHS, NHS2, HPFS) -0.09 0.0112 0.91 [0.89; 0.93] 22.7%
Filled: Chiu 2020 (TCVS) 0.47 0.2322 1.61 [1.02; 2.53] 9.0%
Filled: Chiu 2020 (TCHS) 0.49 0.3685 1.64 [0.80; 3.37] 4.7%
Random effects model 0.94 [0.79; 1.12] 100.0%
Heterogeneity: I^2 = 76%, τ^2 = 0.0350, p < 0.01
0.5
1
2

Supplemental Figure S3. Forest plot of the adjusted relative risk (RR) of plant-based diet and (A) cardiovascular disease and (B) stroke risks with included studies and imputed studies with the trim-and-fill approach.

Supplemental Table S5. Quality assessment of included studies.

	Selection				Comparability	Exposure			
**Cohort studies**	Representativeness of the exposed cohort	Selection of the non exposed cohort	Ascertainment of exposure	Demonstration that outcome of interest was not present at start of study	Comparability	Assessment of outcome	Was follow-up long enough for outcomes to occur	Adequacy of follow up of cohorts	**Total**
Crowe 2013		★	★	★	★★	★	★	★	**8**
Judd 2013	★	★	★	★	★★	★	★	★	**9**
Shikany 2015	★	★	★	★	★★	★	★	★	**9**
Satija 2017 (NHS)		★	★	★	★★	★	★	★	**8**
Satija 2017 (NHS2)		★	★	★	★★	★	★	★	**8**
Satija 2017 (HPFS)		★	★	★	★★	★	★	★	**8**
Kim 2019	★	★	★	★	★★	★	★		**8**
Tong 2019		★	★	★	★★	★	★		**7**
Chiu 2020		★	★	★	★★	★	★		**7**
Heianza 2020	★	★	★	★	★★	★	★		**8**
Petermann-Rocha 2020	★	★	★	★	★★	★	★	★	**9**

Shan 2020	★	★	★	★★	★	★	★	**8**

(A)

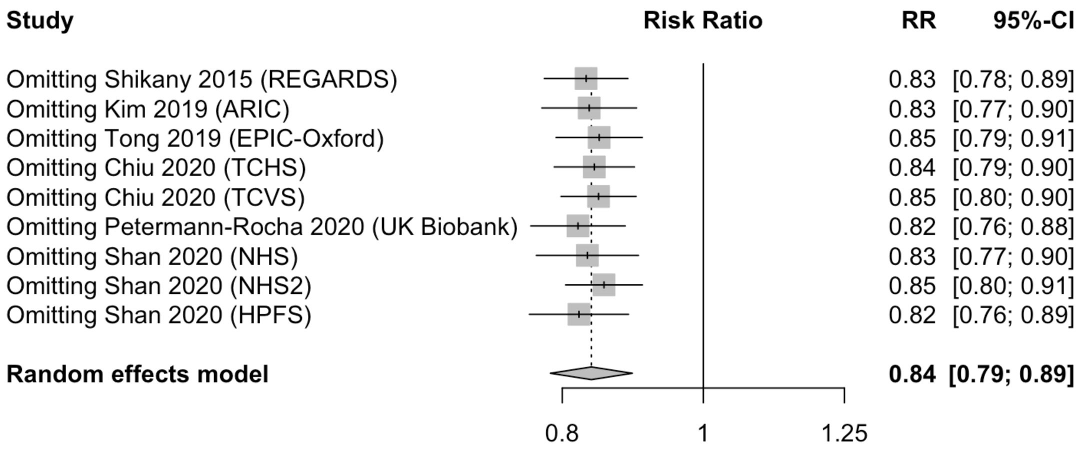

(B)

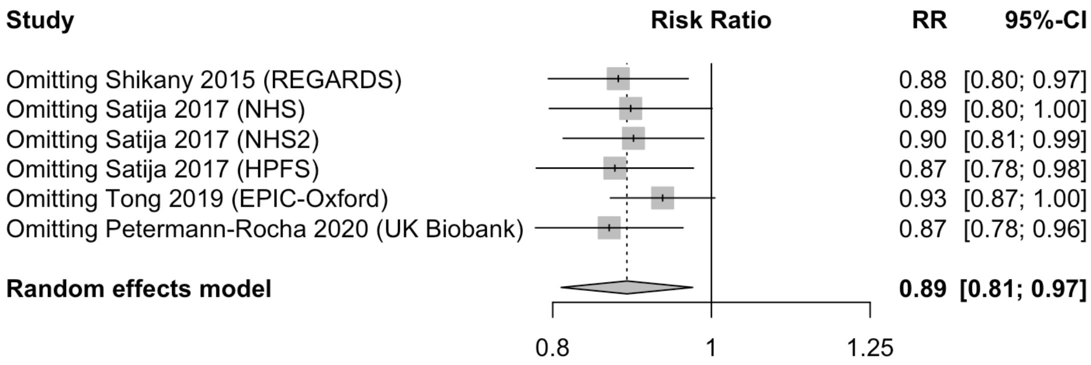

(C)

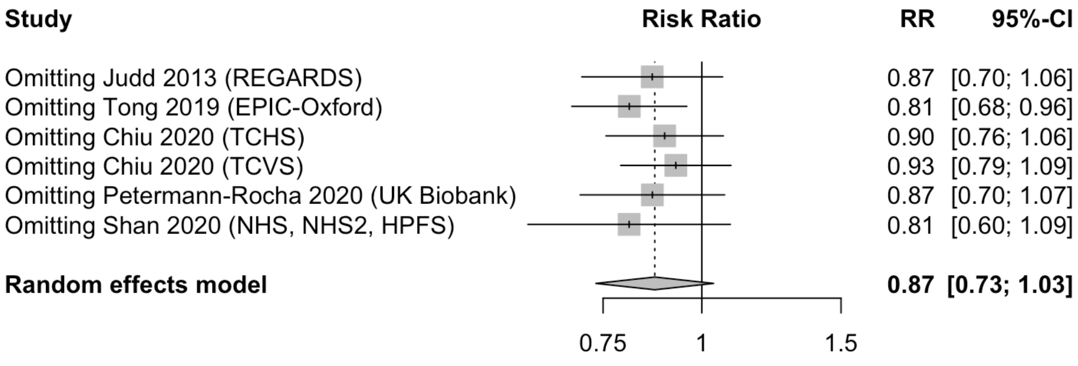

Supplemental Figure S4. Forest plot of the adjusted relative risk (RR) of (A) cardiovascular disease, (B) coronary heart disease and (C) stroke risks for highest versus lowest adherence to plant-based dietary patterns with sensitivity analyses.
